# Altered intra- and inter-network connectivity in autism spectrum disorder

**DOI:** 10.18632/aging.205913

**Published:** 2024-06-10

**Authors:** Rui Zhou, Chenhao Sun, Mingxiang Sun, Yudi Ruan, Weikai Li, Xin Gao

**Affiliations:** 1 School of Zhang Jian, Nantong University, Nantong, China; 2 College of Mathematics and Statistics, Chongqing Jiaotong University, Chongqing, China; 3 Department of Radiology, Rugao Jian’an Hospital, Nantong, China; 4 Shanghai Universal Medical Imaging Diagnostic Center, Shanghai, China; 5 Hubei Province Key Laboratory of Molecular Imaging, Wuhan, China

**Keywords:** autism spectrum disorder, inter-network connectivity, intra-network connectivity, support vector machine, functional brain network

## Abstract

Objective: A neurodevelopmental illness termed as the autism spectrum disorder (ASD) is described by social interaction impairments. Previous studies employing resting-state functional imaging (rs-fMRI) identified both hyperconnectivity and hypoconnectivity patterns in ASD people. However, specific patterns of connectivity within and between networks linked to ASD remain largely unexplored.

Methods: We utilized a meticulously selected subset of high-quality data, comprising 45 individuals diagnosed with ASD and 47 HCs, obtained from the ABIDE dataset. The pre-processed rs-fMRI time series signals were partitioned into ninety regions of interest. We focused on eight intrinsic connectivity networks and further performed intra- and inter-network analysis. Finally, support vector machine was used to discriminate ASD from HC.

Results: Through different sparsities, ASD exhibited significantly decreased intra-network connectivity within default mode network and dorsal attention network, increased connectivity between limbic network and subcortical network, and decreased connectivity between default mode network and limbic network. Using the classifier trained on altered intra- and inter-network connectivity, multivariate pattern analyses classified the ASD from HC with 71.74% accuracy, 70.21% specificity and 75.56% sensitivity in 10% sparsity of functional connectivity.

Conclusions: ASD showed characteristic reorganization of the brain networks and this provided new insight into the underlying process of the functional connectome dysfunction in ASD.

## INTRODUCTION

ASD is a diverse neurodevelopmental illness described by the existence of repetitive and limited interests, and challenges in social communication [1–3]. Earlier research suggested that the symptoms associated with ASD were explained by the essential hypothesis of abnormal neuronal connectivity [1, 4, 5]. However, studies investigating ASD individuals regarding alterations in functional connectivity via the rs-fMRI data have yielded inconsistent findings. Some studies have reported increased hyperconnectivity, while others have observed decreased hyperconnectivity in participants with ASD [6, 7].

Utilizing neuroimaging techniques can contribute to deepening our comprehension of the biological components associated with ASD, complementing behavioral analysis in this domain. Moreover, it holds the capability to identify neural biomarkers for early detection, prognosis, and treatment purposes. In humans, intrinsic functional connectivity serves as an *in vivo* technique of connectivity [8]. The brain functions as a complex network linked by functional connectivity that allows information to be segregated and integrated efficiently at low wiring costs [9]. Numerous investigations have identified disparities in functional connectivity among typically developing controls. Preliminary studies primarily aimed at the connectivity of DMN (default mode network), salience network and executive control network [10, 11]. The anterior-posterior hypoconnectivity, specifically between parietal and frontal DMN regions, best describe the DMN connectivity [12–14]. This has been concluded in several studies through independent component analysis, voxel-wise whole brain analyses, and seed-based analysis. Additional research revealed decreases in brain areas linked to social behavior, language, and communication that are connected to the limbic system [15, 16]. However, Anderson et al. discovered that individuals with ASD exhibited increased connectivity within the DMN, as well as heightened synchronization between the DMN and networks associated with attention [17]. Another study explored the three large-scale networks (DMN, executive control network and salience network) simultaneously using resting-state functional MRI in children and adolescents with ASD. They found that the DMN and executive control network had age-related over-connectivity in young children with ASD but not in adolescents with ASD. This may reflect delayed network segregation in ASD [18] Crucially, several neuroimaging works on ASD have independently targeted the higher-order cortico-cortical functional connectivity. Limited research works have studied the atypical of intra- and inter-network connectivity patterns when considering the entire network as a whole.

The difficulty in synthesizing current literature on ASD functional connectivity arises from the potential for varying analysis approaches to yield conflicting findings. The study herein was focused to explore characteristics and scope of functional disparities within and between networks, utilizing the commonly employed Yeo-network atlas in individuals with ASD. This approach was employed to investigate the similarities and differences in large-scale brain networks between typically developing individuals and ASD diagnosed individuals of similar age. The study findings can improve the insight to neurophysiological basis of ASD, as well as facilitating the development of more precise diagnostic markers and targeted therapeutic strategies.

## MATERIALS AND METHODS

### Participants

In this investigation, we utilized the identical dataset sourced from New York University Langone Medical Center as per the earlier research [4, 19]. This dataset was also available in a previously described Autism Brain Imaging Data Exchange initiative (ABIDE I and II; http://fcon_1000.projects.nitrc.org/indi/abide). All individuals involved in the study were given written consent before participating. The sample consisted of 92 children aged 7 to 15, with 45 diagnosed for ASD and 47 serving as HCs. Diagnosis for those with ASD was determined by a clinical team employing ADOS (Autism Diagnostic Observation Schedule) [20]. The subjects enrolled in research met requirements for ASD according to ADI-R (Autism Diagnostic Interview-Revised), following guidelines defined in Diagnostic and Statistical Manual of Mental Disorders, 4th Edition (DSM-IV) [21]. The domains under investigation encompass repetitive or restricted behaviors, language and communication skills, and reciprocal social interactions. The Wechsler Intelligence Scale for Children-III (WISC III), Wechsler Adult Intelligence Scale-III (WAIS III), and/or Wechsler Abbreviated Scale of Intelligence (WASI) were employed to assess Full-scale Intelligence Quotient (FIQ). Participants with any other neuropsychological or neurological disorders were excluded from all groups. The control group had people with no mental disorders’ history who were carefully matched in terms of age and gender to the ASD group.

### Acquisition of fMRI data

The rs-fMRI data were attained by employing 3 T scanners manufactured by Siemens. Throughout the 6-minute rs-fMRI scan, participants were instructed to maintain a state of relaxation while fixating their gaze on a centrally positioned white cross displayed on a screen having black background. Gradient-echo EPI (echoplanar imaging) sequence included flip angle = 90°, echo time = 15 ms, repetition time = 2000 ms, 180 volumes, 33 slices and voxel size = 3.0 × 3.0 × 4.0 mm^3^.

### Preprocessing of neuroimaging data

DPABI v4.1^1^ and SPM12^2^ were utilized to preprocess the rs-fMRI data. The Wellcome Trust Centre for Neuroimaging at University College London provided this software package. The first ten time points were excluded to ensure signal equilibrium. Subsequent preprocessing steps involved correcting for head functional images to Montreal Neurological Institute space using SPM12 template. The images were then resampled to isotropic voxels measuring 3mm × 3mm × 3mm and further smoothed using a Gaussian kernel. To mitigate the effects of gradual signal drift and unwanted noise at different frequencies, we employed temporal bandpass filtering and linear detrending techniques (0.01 - 0.1 Hz). Furthermore, we conducted regression analysis to eliminate confounding factors such as Friston-24 head motion parameters, cerebrospinal fluid signals, and white matter signals from BOLD (blood oxygen level-dependent) time series data across all voxels.

### Inter- and intra-network connectivity analysis

**Figure 1:**
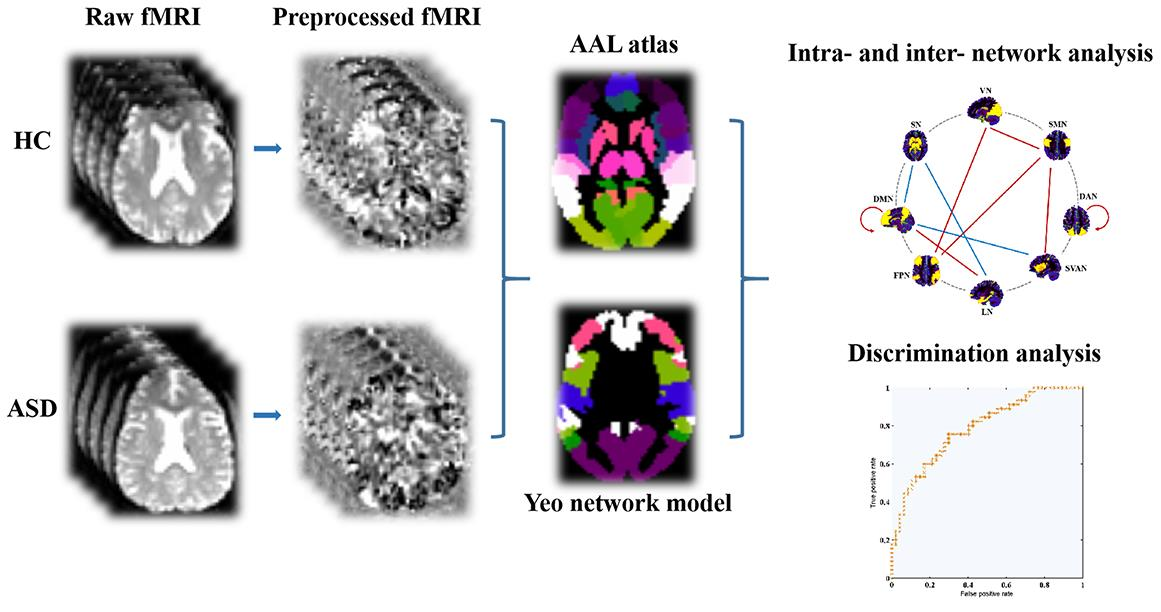
**The fMRI data preprocessing and network analysis depicted via flow diagram.** Abbreviations: AAL, automated anatomical labeling; fMRI, functional magnetic resonance imaging; ASD, autism spectrum disorder; HC, healthy controls.

From AAL atlas, which provides abbreviations for nodes as listed in Supplementary Table 1, rs-fMRI time series signals were divided into 90 ROIs (regions of interest) after undergoing preprocessing. We focused on eight intrinsic connectivity networks including subcortical network (SN) and seven function networks [22]. The 8 canonical neural networks included SN, salience/ventral attention network (SVAN), dorsal attention network (DAN), somatomotor network (SMN), DMN, frontoparietal network (FPN), visual network (VN), and limbic network (LN). Each of the eight networks comprised multiple regions of interest (ROIs). This procedure relied on matching the voxels’ label from Yeo-7 network atlas to AAL atlas. In terms of subject-level analysis, Pearson’s correlation coefficient was calculated to assess the functional connectivity between 2 cortical ROIs through average fMRI time series data from these ROIs. Subsequently, Fisher’s r-to-z transformation occurred for each correlation coefficient. A matrix representing subject-level z-scores for functional connectivity was then constructed, encompassing 90 ROIs from eight distinct functional networks. Note that the negative correlations took only a very small portion and had the ambiguous biological explanation, here we only focused on the positive connections. To facilitate further statistical analyses, we computed the mean z-scores for both intra-network connectivity (e.g., DMN, FPN, LN, VN) and inter-network connectivity (DMN, FPN, DMN, LN, FPN, VN). The GRETNA toolbox^3^ was applied for intra- and inter-network analysis. Considering the potential impact of sparsity threshold on outcomes, we conducted network analysis using sparsity thresholds of 10%, 20%, and 30% consecutively. The flow diagram of network analysis was described in Figure 1.

### Statistical analysis

Statistical Package for Social Sciences (SPSS, V22^4^) was utilized to compare the neuroimaging and demographic properties of HC and ASD groups. A two-sample t-test evaluated age disparities between groups, while gender variances were analyzed using a chi-square (χ^2^) test. Additionally, group distinctions in intra- and inter-network connectivity were examined utilizing a two-sample t-test (uncorrected p-value).

### Support vector machine (SVM)

Disease classification is made by SVM analysis. There are 3 stages in SVM protocol: feature selection, classifier training, and prediction [23]. SVM initiates by selecting relevant features to construct a reproducing kernel hilbert space (RKHS) for classification purposes. In this study, we specifically identified statistically significant features linked to inter- and intra-network connectivity to be utilized in our SVM model. Subsequently, the SVM algorithm proceeds to train a classifier by constructing an optimal hyperplane that effectively discriminates between different classes classifier which is utilized for predicting the class label of any newly introduced sample. The SVM analysis was performed using the LIBSVM toolkit^5^ in this study. We utilized leave-one-out cross-validation (LOOCV) to mitigate the issue of a small sample size for assessing mean accuracy rate in distinguishing individuals diagnosed with ASD from HC [24, 25]. The classifier can be assessed through area under the receiver operating characteristic (ROC) curve, specificity, and sensitivity based on cross-validation results. It is worth noting that specificity reflects the accuracy in correctly predicting healthy control subjects, while sensitivity represents the accuracy in correctly identifying individuals with ASD.

## RESULTS

### Clinical and demographic characteristics

No statistically significant difference was found in FIQ (p = 0.05), gender (p = 0.21), and age (p = 0.78) as shown in Table 1. In ASD group, the mean score of ADI-R was 32.2 ± 14.3 and the mean score of ADOS was 13.7 ± 5.0.

**Table 1:** Clinical and demographic data in ASD and HC.

**Items**	**HC (n = 47)**	**ASD (n = 45)**	***p*-value**
Age (years)	11.0 ± 2.3	11.1 ± 2.3	0.78b
Gender (male/female)	36/11	36/9	0.21a
FIQ (mean ± SD)	113.3 ± 14.1	106.8 ± 17.4	0.05
ADI-R (mean ± SD)	--	32.2 ± 14.3#	--
ADOS (mean ± SD)	--	13.7 ± 5.0	--

Values given as mean ± standard deviation (SD).

The p-value was attained by χ2 test, b the p-value was attained by two-sample t-tests.

Abbreviations: ADOS, Autism Diagnostic Observation Schedule; ADI-R, Autism Diagnostic Interview-Revised; FIQ, Full-scale Intelligence Quotient; HC, healthy controls; ASD, autism spectrum disorders; ^#^Two patients had no ADI-R score.

### Altered intra- and inter-network connectivity in ASD

**Figure 2:**
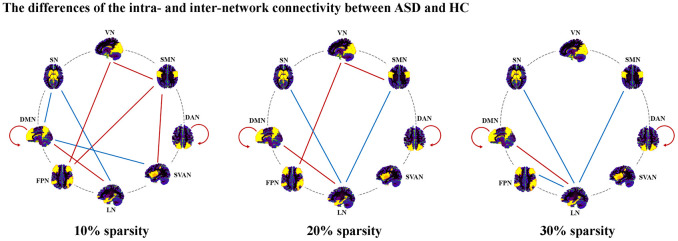
**Altered inter- and intra-network connectivity in ASD.** Abbreviations: DAN, dorsal attention network; SVAN, salience/ventral attention network; DMN, default mode network; FPN, frontoparietal network; SMN, somatomotor network; VN, visual network; LN, limbic network; SN, subcortical network; ASD, autism spectrum disorder; HC, healthy controls.

In 10% sparsity (Figure 2 and Table 2), we found significant decreases in intra-network connectivity within DMN (t = -2.37, p = 0.020) and DAN (t = -2.61, p = 0.011) in ASD. In addition, ASD depicted reduced inter-network connectivity in DMN-LN (t = -2.34, p = 0.021), SMN-SVAN (t = -2.60, p = 0.011), SMN-FPN (t = -2.07, p = 0.041), VN-FPN (t = -2.02, p = 0.046), and VN-SMN (t = -2.05, p = 0.043), while ASD showed significantly increased inter-network connectivity in LN-SN (t = 4.21, p < 0.001), SVAN-DMN (t = 2.18, p = 0.032) and DMN-SN (t = 2.03, p = 0.045). Notably, only the increased inter-network connectivity of LN-SN in the ASD group could survive multiple comparison corrections (p < 0.05, Bonferroni corrected).

**Table 2:** The significant difference of intra- and inter-network analysis.

**Sparsity**	**Connection**	**HC**	**ASD**	***t* value**	**p-value**
10%	Intra-network				
	DAN	0.73 ± 0.25	0.90 ± 0.35	-2.61	0.011
	DMN	22.96 ± 6.39	26.29 ± 7.06	-2.37	0.020
	Inter-network				
	VN-SMN	0.27 ± 0.60	1.19 ± 2.94	-2.05	0.043
	VN-FPN	0.14 ± 0.35	0.48 ± 1.07	-2.02	0.046
	SMN-SVAN	12.53 ± 5.55	15.77 ± 6.35	-2.60	0.011
	SMN-FPN	2.62 ± 1.80	3.54 ± 2.38	-2.07	0.041
	SVAN-DMN	3.96 ± 2.78	2.90 ± 1.77	2.18	0.032
	LN-DMN	14.48 ± 5.37	17.20 ± 5.73	-2.34	0.021
	LN-SN	12.04 ± 5.80	7.92 ± 3.32	4.21	<0.001
	DMN-SN	3.32 ± 3.10	2.11 ± 2.62	2.03	0.045
20%	Intra-network				
	DAN	0.89 ± 0.32	1.10 ± 0.43	-2.65	0.009
	DMN	32.03 ± 6.97	36.71 ± 8.29	-2.92	0.004
	Inter-network				
	VN-SMN	1.89 ± 1.99	3.93 ± 6.57	-2.00	0.049
	VN-FPN	0.85 ± 1.18	1.64 ± 2.40	-2.00	0.049
	SMN-LN	11.93 ± 6.53	9.14 ± 4.41	2.41	0.018
	LN-DMN	25.84 ± 7.21	29.72 ± 7.58	-2.51	0.014
	LN-SN	19.47 ± 7.85	13.93 ± 4.29	4.23	<0.001
30%	Intra-network				
	DAN	1.01 ± 0.31	1.67 ± 0.41	-2.08	0.040
	DMN	36.69 ± 7.46	41.51 ± 8.62	-2.87	0.005
	Inter-network				
	SMN-LN	16.39 ± 7.29	13.71 ± 5.30	2.02	0.046
	LN-FPN	9.80 ± 4.73	8.06 ± 3.17	2.09	0.040
	LN-DMN	33.21 ± 7.69	37.46 ± 8.32	-2.54	0.013
	LN-SN	23.39 ± 8.26	18.32 ± 5.22	3.53	0.001

Abbreviations: DAN, dorsal attention network; SVAN, salience/ventral attention network; DMN, default mode network; FPN, frontoparietal network; SMN, somatomotor network; VN, visual network; LN, limbic network; SN, subcortical network; HC, healthy controls; ASD, autism spectrum disorders.

In 20% sparsity (Figure 2 and Table 2), significant decreases in intra-network connectivity within DMN (t = -2.92, p = 0.004) and DAN (t = -2.65, p = 0.009) in ASD were found. We found significantly decreased inter-network connectivity in LN-DMN (t = -2.51, p = 0.014), VN-FPN (t = -2.00, p = 0.049), and VN-SMN (t = -2.00, p = 0.049) in ASD. In addition, ASD showed significantly increased inter-network connectivity in LN-SN (t = 4.23, p < 0.001) and SMN-LN (t = 2.41, p = 0.018). Notably, only the increased inter-network connectivity of LN-SN in the ASD group could survive multiple comparison corrections (p < 0.05, Bonferroni corrected).

In 30% sparsity (Figure 2 and Table 2), we found significant decreases in intra-network connectivity within DMN (t = -2.87, p = 0.005) and DAN (t = -2.08, p = 0.040) in ASD. Significantly decreased inter-network connectivity in LN-DMN (t = -2.54, p = 0.013) were found in ASD. In addition, ASD showed significantly increased inter-network connectivity in LN-SN (t = 3.53, p = 0.001), LN-FPN (t = 2.09, p = 0.040), and SMN-LN (t = 2.02, p = 0.046). Notably, only the increased inter-network connectivity of LN-SN in the ASD group could survive multiple comparison corrections (p < 0.05, Bonferroni corrected).

Through these sparsities, ASD exhibited increased inter-network connectivity in LN-SN, decreased inter-network connectivity in LN-DMN, and decreased intra-network connectivity within DAN and DMN.

Discriminative analysis based on changed inter- and intra-network connectivity.

**Figure 3:**
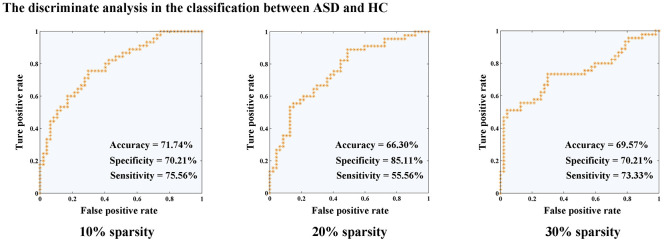
**Discriminate analysis for the classification between ASD and HC.** Abbreviations: HC, healthy controls; ASD, autism spectrum disorder.

Classifier trained on modified inter- and intra-network connectivity, along with the LOOCV (Figure 3), achieved a classification accuracy of 71.74%, specificity of 70.21%, and sensitivity of 75.56% in functional connectivity sparsity of 10%. In the case of 20% sparsity, the classification performance yielded 66.30% accuracy, 85.11% specificity, and 55.56% sensitivity. Furthermore, at a sparsity level of 30%, the classification performance resulted in an accuracy rate of 69.57%, specificity rate of 70.21%, and sensitivity rate of 73.33%.

## DISCUSSION

The rs-fMRI imaging data was utilized in this research for creating a functional brain network and investigated the modified connectivity patterns within and between networks in ASD diagnosed individuals. Furthermore, machine learning methodologies were used to investigate diagnostic value of this altered pattern in ASD. Our results revealed that ASD showed characteristic reorganization of the brain networks referring to increased inter-network connectivity in LN-SN, decreased inter-network connectivity in LN-DMN, and decreased intra-network connectivity within DAN and DMN. This altered pattern could serve as a potential biomarker for ASD.

Earlier works utilizing rs-fMRI in human brains have identified several prominent networks that exhibit intrinsic connectivity and are associated with visual, motor, auditory, memory, and executive processes. Regarding ASD, three fundamental neurocognitive networks have received considerable attention: the salience network, Frontoparietal Network (FPN), and Default Mode Network (DMN) [26]. The DMN primarily encompasses regions such as inferior parietal cortex, precuneus, medial prefrontal cortex, and posterior cingulate cortex (PCC). Extensive work has been conducted to investigate DMN connectivity in relation to elucidating the hypothesis of Theory of Mind (ToM) regarding ASD pathogenesis [27]. ToM hypothesis relates to the ability of a person to recognize subjective mental states of others, including intentions and perspective, and if scenario is hypothetical or real [28]. ToM hypothesis pertains to an individual’s capacity to comprehend subjective cognitive states of others, encompassing intentions and perspectives, regardless of whether situation is hypothetical or actual [20]. Additionally, the hypothesis of ToM proposes that ASD diagnosed individuals, a deficient or underdeveloped ToM, that hinders their capacity to deduce mental conditions of others - a fundamental aspect of social interaction [29].

In our study, ASD showed significant decreased intra-network connectivity within DMN. Previous works have investigated the degree of ToM impairment in ASD and observed the hypoconnectivity in DMN to evaluate the possible neural basis for the theory [27, 30, 31]. Assaf et al. investigated role of altered functional connectivity within DMN through independent component analysis [30]. ASD individuals exhibited reduced connectivity between medial prefrontal cortex and precuneus, and other core areas of DMN, which aligns with our findings. Notably, we observed an inverse relation between connectivity of these regions, and extreme communication and social declines in patients, as assessed by Autism Diagnostic Observational Schedule and Social Responsiveness Scale [32]. Another study based on PCC-seed analysis also demonstrated that weaker connectivity within the DMN linked to specific ASD impairments [33]. However, Anderson and colleagues proposed hyperconnectivity within DMN and enhanced inter-network synchrony between attentional networks and DMN in ASD [28]. Redcay and colleagues examined 4 discrete networks (FPN, cerebellar network, cingulo-opercular network, and DMN) in ASD based on graph theory. Minimal differences were found in the adolescent males between ASD and controls [34]. The variations in results can be attributed to heterogeneous nature of ASD, fMRI scanning parameters and analysis strategy. In addition, the decreased connectivity within DAN was also found in our study. DAN comprises vital nodes in frontal eye fields and bilateral intraparietal sulcus, and regulates the intentional, goal-oriented top down “endogenous” attention [35]. Studies on attention in individuals with ASD have identified varying degrees of attention deficits. Pruett and colleagues conducted an attention orienting task, which depicted that autism children had increased eye movements and slower response times during visual orientation in comparison to typically developing individuals [36, 37]. Fitzgerald and his team employed psychophysical interaction analysis for investigating the impact of tasks on brain functional connectivity, evaluating the degree of synchronization between different regions while performing the task [38]. Analysis conducted by DAN revealed a decrease in coherence among brain regions responsible for regulating goal-oriented, internally-driven attention in individuals with ASD. The outcomes were in accordance with our ASD results.

In this study, we found that ASD also showed increased inter-network connectivity in LN-SN in comparison to the adolescents developed typically. Hyperconnectivity of subcortical regions in ASD individuals was initially demonstrated by Di Martino and colleagues through conducted on school-age children [39]. The increased connectivity was found between heteromodal associative and limbic cortex and the striatum. Another study examining subcortical connectivity also found hyperconnectivity between frontal cortex and striatum [40]. The present study has observed an elevated level of hyperconnectivity between primary sensory regions and the salience network in individuals diagnosed with ASD. Furthermore, it was found that the extent of this heightened connectivity is positively linked to symptom severity, demonstrating that impaired sensory connectivity can contribute to the manifestation of autistic behaviors [41]. These findings provide evidence supporting an emerging hypothesis that proposes reduced segregation among functional networks of ASD individuals and enhanced networks connectivity. The presence of increased diffuse connectivity allows for the coexistence of reports on both hypo- and hyper-connectivity, as it attenuates traditionally strong connections while introducing novel connections.

Comprehending and deciphering brain findings related to ASD poses intricate difficulties. Various constraints are associated with this research. First, only a cross-sectional design was employed in this study and conducted on small sample size of individuals with ASD. To strengthen our outcomes, we anticipate expanding sample size and conducting a multicenter longitudinal follow-up study, which is imperative for further investigation. Second, we exclusively focused on studying functional networks. The integration of structural and functional analyses represents a biologically sound approach that confers methodological advantages in the field of neuroimaging research on ASD. Employing multimodal imaging analysis would enhance our thorough perception of relationship between disease symptoms and imaging biomarkers in ASD individuals. Third, future investigations aiming to utilize connectivity-based measurements for preclinical goals, like establishing biologically-grounded ASD subgroups or predicting treatment response, must be taken into consideration.

## CONCLUSIONS

Our research findings have unveiled unique reorganization patterns in brain networks of ASD people, providing novel insights into the underlying mechanism behind functional connectome dysfunction observed in ASD.

## Supplementary Material

Supplementary Table 1

## Footnotes

1
http://rfmri.org/dpabi/

2
http://www.fil.ion.ucl.ac.uk/spm/soft-ware/spm12

3
http://www.nitrc.org/projects/gretna/

4
http://www.spss.com/

5
http://www.csie.ntu.edu.tw/~cjlin/libsvm/

## References

[1] Cooper R. Diagnosing the diagnostic and statistical manual of mental disorders. 2018. Routledge. 10.4324/9780429473678

[2] Lord C, Brugha TS, Charman T, Cusack J, Dumas G, Frazier T, Jones EJ, Jones RM, Pickles A, State MW, Taylor JL, Veenstra-VanderWeele J. Autism spectrum disorder. Nat Rev Dis Primers. 2020; 6:5. 10.1038/s41572-019-0138-4 31949163 PMC8900942

[3] Yang J, Xu X, Sun M, Ruan Y, Sun C, Li W, Gao X. Towards an accurate autism spectrum disorder diagnosis: multiple connectome views from fMRI data. Cereb Cortex. 2024; 34:bhad477. 10.1093/cercor/bhad477 38100334

[4] Meyer PT, Frings L, Rücker G, Hellwig S. ^18^F-FDG PET in Parkinsonism: Differential Diagnosis and Evaluation of Cognitive Impairment. J Nucl Med. 2017; 58:1888–98. 10.2967/jnumed.116.186403 28912150

[5] Belmonte MK, Allen G, Beckel-Mitchener A, Boulanger LM, Carper RA, Webb SJ. Autism and abnormal development of brain connectivity. J Neurosci. 2004; 24:9228–31. 10.1523/JNEUROSCI.3340-04.2004 15496656 PMC6730085

[6] Hull JV, Dokovna LB, Jacokes ZJ, Torgerson CM, Irimia A, Van Horn JD. Resting-State Functional Connectivity in Autism Spectrum Disorders: A Review. Front Psychiatry. 2017; 7:205. 10.3389/fpsyt.2016.00205 28101064 PMC5209637

[7] Li W, Wang Z, Zhang L, Qiao L, Shen D. Remodeling Pearson’s Correlation for Functional Brain Network Estimation and Autism Spectrum Disorder Identification. Front Neuroinform. 2017; 11:55. 10.3389/fninf.2017.00055 28912708 PMC5583214

[8] Minshew NJ, Keller TA. The nature of brain dysfunction in autism: functional brain imaging studies. Curr Opin Neurol. 2010; 23:124–30. 10.1097/WCO.0b013e32833782d4 20154614 PMC2975255

[9] Bassett DS, Bullmore ET. Small-World Brain Networks Revisited. Neuroscientist. 2017; 23:499–516. 10.1177/1073858416667720 27655008 PMC5603984

[10] Broyd SJ, Demanuele C, Debener S, Helps SK, James CJ, Sonuga-Barke EJ. Default-mode brain dysfunction in mental disorders: a systematic review. Neurosci Biobehav Rev. 2009; 33:279–96. 10.1016/j.neubiorev.2008.09.002 18824195

[11] Uddin LQ, Dajani DR, Voorhies W, Bednarz H, Kana RK. Progress and roadblocks in the search for brain-based biomarkers of autism and attention-deficit/hyperactivity disorder. Transl Psychiatry. 2017; 7:e1218. 10.1038/tp.2017.164 28892073 PMC5611731

[12] Washington SD, Gordon EM, Brar J, Warburton S, Sawyer AT, Wolfe A, Mease-Ference ER, Girton L, Hailu A, Mbwana J, Gaillard WD, Kalbfleisch ML, VanMeter JW. Dysmaturation of the default mode network in autism. Hum Brain Mapp. 2014; 35:1284–96. 10.1002/hbm.22252 23334984 PMC3651798

[13] Wiggins JL, Peltier SJ, Ashinoff S, Weng SJ, Carrasco M, Welsh RC, Lord C, Monk CS. Using a self-organizing map algorithm to detect age-related changes in functional connectivity during rest in autism spectrum disorders. Brain Res. 2011; 1380:187–97. 10.1016/j.brainres.2010.10.102 21047495 PMC3050117

[14] Chen L, Chen Y, Zheng H, Zhang B, Wang F, Fang J, Li Y, Chen Q, Zhang S. Changes in the topological organization of the default mode network in autism spectrum disorder. Brain Imaging Behav. 2021; 15:1058–67. 10.1007/s11682-020-00312-8 32737824

[15] Gotts SJ, Simmons WK, Milbury LA, Wallace GL, Cox RW, Martin A. Fractionation of social brain circuits in autism spectrum disorders. Brain. 2012; 135:2711–25. 10.1093/brain/aws160 22791801 PMC3437021

[16] Fu L, Li C, Li Y, Cheng X, Cui X, Jiang J, Ding N, Fang H, Tang T, Ke X. Heritability of abnormalities in limbic networks of autism spectrum disorder children: Evidence from an autism spectrum disorder twin study. Autism Res. 2022; 15:628–40. 10.1002/aur.2686 35212461

[17] Anderson JS, Nielsen JA, Ferguson MA, Burback MC, Cox ET, Dai L, Gerig G, Edgin JO, Korenberg JR. Abnormal brain synchrony in Down Syndrome. Neuroimage Clin. 2013; 2:703–15. 10.1016/j.nicl.2013.05.006 24179822 PMC3778249

[18] Abbott AE, Nair A, Keown CL, Datko M, Jahedi A, Fishman I, Müller RA. Patterns of Atypical Functional Connectivity and Behavioral Links in Autism Differ Between Default, Salience, and Executive Networks. Cereb Cortex. 2016; 26:4034–45. 10.1093/cercor/bhv191 26351318 PMC5027998

[19] Peng L, Chen Z, Gao X. Altered rich-club organization of brain functional network in autism spectrum disorder. Biofactors. 2023; 49:612–9. 10.1002/biof.1933 36785880

[20] Lord C, Risi S, Lambrecht L, Cook EH Jr, Leventhal BL, DiLavore PC, Pickles A, Rutter M. The autism diagnostic observation schedule-generic: a standard measure of social and communication deficits associated with the spectrum of autism. J Autism Dev Disord. 2000; 30:205–23. 11055457

[21] Lord C, Rutter M, Le Couteur A. Autism Diagnostic Interview-Revised: a revised version of a diagnostic interview for caregivers of individuals with possible pervasive developmental disorders. J Autism Dev Disord. 1994; 24:659–85. 10.1007/BF02172145 7814313

[22] Yeo BT, Krienen FM, Sepulcre J, Sabuncu MR, Lashkari D, Hollinshead M, Roffman JL, Smoller JW, Zöllei L, Polimeni JR, Fischl B, Liu H, Buckner RL. The organization of the human cerebral cortex estimated by intrinsic functional connectivity. J Neurophysiol. 2011; 106:1125–65. 10.1152/jn.00338.2011 21653723 PMC3174820

[23] Li W, Qiao L, Zhang L, Wang Z, Shen D. Functional Brain Network Estimation With Time Series Self-Scrubbing. IEEE J Biomed Health Inform. 2019; 23:2494–504. 10.1109/JBHI.2019.2893880 30668484 PMC6904893

[24] Li W, Geng C, Chen S, Leave Zero Out: Towards a No-Cross-Validation Approach for Model Selection. arXiv preprint arXiv:2012.13309. 2020.

[25] Li W, Zhang L, Qiao L, Shen D. Toward a Better Estimation of Functional Brain Network for Mild Cognitive Impairment Identification: A Transfer Learning View. IEEE J Biomed Health Inform. 2020; 24:1160–8. 10.1109/JBHI.2019.2934230 31403449 PMC7285887

[26] Chen YY, Uljarevic M, Neal J, Greening S, Yim H, Lee TH. Excessive Functional Coupling With Less Variability Between Salience and Default Mode Networks in Autism Spectrum Disorder. Biol Psychiatry Cogn Neurosci Neuroimaging. 2022; 7:876–84. 10.1016/j.bpsc.2021.11.016 34929345

[27] Lawrence KE, Hernandez LM, Bookheimer SY, Dapretto M. Atypical longitudinal development of functional connectivity in adolescents with autism spectrum disorder. Autism Res. 2019; 12:53–65. 10.1002/aur.1971 30375176 PMC6325013

[28] Fakhoury M. Autistic spectrum disorders: A review of clinical features, theories and diagnosis. Int J Dev Neurosci. 2015; 43:70–7. 10.1016/j.ijdevneu.2015.04.003 25862937

[29] Premack D, Woodruff G. Chimpanzee problem-solving: a test for comprehension. Science. 1978; 202:532–5. 10.1126/science.705342 705342

[30] Assaf M, Jagannathan K, Calhoun VD, Miller L, Stevens MC, Sahl R, O’Boyle JG, Schultz RT, Pearlson GD. Abnormal functional connectivity of default mode sub-networks in autism spectrum disorder patients. Neuroimage. 2010; 53:247–56. 10.1016/j.neuroimage.2010.05.067 20621638 PMC3058935

[31] Weng SJ, Wiggins JL, Peltier SJ, Carrasco M, Risi S, Lord C, Monk CS. Alterations of resting state functional connectivity in the default network in adolescents with autism spectrum disorders. Brain Res. 2010; 1313:202–14. 10.1016/j.brainres.2009.11.057 20004180 PMC2818723

[32] Padmanabhan A, Lynch CJ, Schaer M, Menon V. The Default Mode Network in Autism. Biol Psychiatry Cogn Neurosci Neuroimaging. 2017; 2:476–86. 10.1016/j.bpsc.2017.04.004 29034353 PMC5635856

[33] Lau WK, Leung MK, Zhang R. Hypofunctional connectivity between the posterior cingulate cortex and ventromedial prefrontal cortex in autism: Evidence from coordinate-based imaging meta-analysis. Prog Neuropsychopharmacol Biol Psychiatry. 2020; 103:109986. 10.1016/j.pnpbp.2020.109986 32473190

[34] Redcay E, Moran JM, Mavros PL, Tager-Flusberg H, Gabrieli JD, Whitfield-Gabrieli S. Intrinsic functional network organization in high-functioning adolescents with autism spectrum disorder. Front Hum Neurosci. 2013; 7:573. 10.3389/fnhum.2013.00573 24062673 PMC3777537

[35] Kincade JM, Abrams RA, Astafiev SV, Shulman GL, Corbetta M. An event-related functional magnetic resonance imaging study of voluntary and stimulus-driven orienting of attention. J Neurosci. 2005; 25:4593–604. 10.1523/JNEUROSCI.0236-05.2005 15872107 PMC6725019

[36] Pruett JR Jr, LaMacchia A, Hoertel S, Squire E, McVey K, Todd RD, Constantino JN, Petersen SE. Social and non-social cueing of visuospatial attention in autism and typical development. J Autism Dev Disord. 2011; 41:715–31. 10.1007/s10803-010-1090-z 20809377 PMC3660145

[37] Cardillo R, Vio C, Mammarella IC. A comparison of local-global visuospatial processing in autism spectrum disorder, nonverbal learning disability, ADHD and typical development. Res Dev Disabil. 2020; 103:103682. 10.1016/j.ridd.2020.103682 32442872

[38] Fitzgerald J, Johnson K, Kehoe E, Bokde AL, Garavan H, Gallagher L, McGrath J. Disrupted functional connectivity in dorsal and ventral attention networks during attention orienting in autism spectrum disorders. Autism Res. 2015; 8:136–52. 10.1002/aur.1430 25428212

[39] Di Martino A, Kelly C, Grzadzinski R, Zuo XN, Mennes M, Mairena MA, Lord C, Castellanos FX, Milham MP. Aberrant striatal functional connectivity in children with autism. Biol Psychiatry. 2011; 69:847–56. 10.1016/j.biopsych.2010.10.029 21195388 PMC3091619

[40] Delmonte S, Gallagher L, O’Hanlon E, McGrath J, Balsters JH. Functional and structural connectivity of frontostriatal circuitry in Autism Spectrum Disorder. Front Hum Neurosci. 2013; 7:430. 10.3389/fnhum.2013.00430 23964221 PMC3734372

[41] Cerliani L, Mennes M, Thomas RM, Di Martino A, Thioux M, Keysers C. Increased Functional Connectivity Between Subcortical and Cortical Resting-State Networks in Autism Spectrum Disorder. JAMA Psychiatry. 2015; 72:767–77. 10.1001/jamapsychiatry.2015.0101 26061743 PMC5008437

