# Supplementary material for: Altered intra- and inter-network connectivity in autism spectrum disorder
Source: Aging (Albany NY). 2024 Jun 10;16(11):10004–15. doi: 10.18632/aging.205913 (PMC11210244; doi:10.18632/aging.205913)
Supplement: Supplementary Table 1 [file aging-16-205913-s001.pdf]

## SUPPLEMENTARY TABLE

**Supplementary Table 1. Brain regions and their abbreviations in AAL atlas.**

| Labels | Abbreviations | Brain regions |
|---|---|---|
| 1 | PrecentralL | Precental gyrus |
| 2 | PrecentralR | Precental gyrus |
| 3 | FrontalSupL | Superior frontal gyrus, dorsolateral |
| 4 | FrontalSupR | Superior frontal gyrus, dorsolateral |
| 5 | FrontalSupOrbL | Superior frontal gyrus, orbital part |
| 6 | FrontalSupOrbR | Superior frontal gyrus, orbital part |
| 7 | FrontalMidL | Middle frontal gyrus |
| 8 | FrontalMidR | Middle frontal gyrus |
| 9 | FrontalMidOrbL | Middle frontal gyrus, orbital part |
| 10 | FrontalMidOrbR | Middle frontal gyrus, orbital part |
| 11 | FrontalInfOperL | Inferior frontal gyrus, opercular part |
| 12 | FrontalInfOperR | Inferior frontal gyrus, opercular part |
| 13 | FrontalInfTriL | Inferior frontal gyrus, triangular part |
| 14 | FrontalInfTriR | Inferior frontal gyrus, triangular part |
| 15 | FrontalInfOrbL | Inferior frontal gyrus, orbital part |
| 16 | FrontalInfOrbR | Inferior frontal gyrus, orbital part |
| 17 | RolandicOperL | Rolandic operculum |
| 18 | RolandicOperR | Rolandic operculum |
| 19 | SuppMotorAreaL | Supplementary motor area |
| 20 | SuppMotorAreaR | Supplementary motor area |
| 21 | OlfactoryL | Olfactory cortex |
| 22 | OlfactoryR | Olfactory cortex |
| 23 | FrontalSupMedialL | Superior frontal gyrus, medial |
| 24 | FrontalSupMedialR | Superior frontal gyrus, medial |
| 25 | FrontalMidOrbL | Superior frontal gyrus, medial orbital |
| 26 | FrontalMidOrbR | Superior frontal gyrus, medial orbital |
| 27 | RectusL | Gyrus rectus |
| 28 | RectusR | Gyrus rectus |
| 29 | InsulaL | Insula |
| 30 | InsulaR | Insula |
| 31 | CingulumAntL | Anterior cingulate and paracingulate gyri |
| 32 | CingulumAntR | Anterior cingulate and paracingulate gyri |
| 33 | CingulumMidL | Median cingulate and paracingulate gyri |
| 34 | CingulumMidR | Median cingulate and paracingulate gyri |
| 35 | CingulumPostL | Posterior cingulate gyrus |
| 36 | CingulumPostR | Posterior cingulate gyrus |
| 37 | HippocampusL | Hippocampus |
| 38 | HippocampusR | Hippocampus |
| 39 | ParaHippocampalL | Parahippocampal gyrus |
| 40 | ParaHippocampalR | Parahippocampal gyrus |
| 41 | AmygdalaL | Amygdala |
| 42 | AmygdalaR | Amygdala |
| 43 | CalcarineL | Calcarine fissure and surrounding cortex |
| 44 | CalcarineR | Calcarine fissure and surrounding cortex |
| 45 | CuneusL | Cuneus |
| 46 | CuneusR | Cuneus |
| 47 | LingualL | Lingual gyrus |
| 48 | LingualR | Lingual gyrus |

| | | |
|---|---|---|
| 49 | OccipitalSupL | Superior occipital gyrus |
| 50 | OccipitalSupR | Superior occipital gyrus |
| 51 | OccipitalMidL | Middle occipital gyrus |
| 52 | OccipitalMidR | Middle occipital gyrus |
| 53 | OccipitalInfL | Inferior occipital gyrus |
| 54 | OccipitalInfR | Inferior occipital gyrus |
| 55 | FusiformL | Fusiform gyrus |
| 56 | FusiformR | Fusiform gyrus |
| 57 | PostcentralL | Postcentral gyrus |
| 58 | PostcentralR | Postcentral gyrus |
| 59 | ParietalSupL | Superior parietal gyrus |
| 60 | ParietalSupR | Superior parietal gyrus |
| 61 | ParietalInfL | Inferior parietal, but supramarginal and angular gyri |
| 62 | ParietalInfR | Inferior parietal, but supramarginal and angular gyri |
| 63 | SupraMarginalL | Supramarginal gyrus |
| 64 | SupraMarginalR | Supramarginal gyrus |
| 65 | AngularL | Angular gyrus |
| 66 | AngularR | Angular gyrus |
| 67 | PrecuneusL | Precuneus |
| 68 | PrecuneusR | Precuneus |
| 69 | ParacentralLobuleL | Paracentral lobule |
| 70 | ParacentralLobuleR | Paracentral lobule |
| 71 | CaudateL | Caudate nucleus |
| 72 | CaudateR | Caudate nucleus |
| 73 | PutamenL | Lenticular nucleus, putamen |
| 74 | PutamenR | Lenticular nucleus, putamen |
| 75 | PallidumL | Lenticular nucleus, pallidum |
| 76 | PallidumR | Lenticular nucleus, pallidum |
| 77 | ThalamusL | Thalamus |
| 78 | ThalamusR | Thalamus |
| 79 | HeschlL | Heschl gyrus |
| 80 | HeschlR | Heschl gyrus |
| 81 | TemporalSupL | Superior temporal gyrus |
| 82 | TemporalSupR | Superior temporal gyrus |
| 83 | TemporalPoleSupL | Temporal pole: superior temporal gyrus |
| 84 | TemporalPoleSupR | Temporal pole: superior temporal gyrus |
| 85 | TemporalMidL | Middle temporal gyrus |
| 86 | TemporalMidR | Middle temporal gyrus |
| 87 | TemporalPoleMidL | Temporal pole: middle temporal gyrus |
| 88 | TemporalPoleMidR | Temporal pole: middle temporal gyrus |
| 89 | TemporalInfL | Inferior temporal gyrus |
| 90 | TemporalInfR | Inferior temporal gyrus |

Abbreviations: AAL, Anatomical Automatic Labeling.
